# The Additive Psychosocial Effects of Binge Eating and Food Insecurity Among Midlife and Older Women

**DOI:** 10.3390/nu17040730

**Published:** 2025-02-19

**Authors:** Lisa Smith Kilpela, Taylur Loera, Salomé Adelia Wilfred, Jessica Salinas, Sabrina E. Cuauro, Carolyn Black Becker

**Affiliations:** 1 Center for Research to Advance Community Health (ReACH Center), Department of Medicine, University of Texas Health Science Center at San Antonio, San Antonio, TX 78229, USA; loerat@uthscsa.edu (T.L.); wilfred@uthscsa.edu (S.A.W.); salinasj11@uthscsa.edu (J.S.); 2 South Texas Veterans Healthcare System, Geriatric Research Education and Clinical Center (GRECC), San Antonio, TX 78229, USA; 3 Department of Psychology, Trinity University, San Antonio, TX 78212, USA; sc235@rice.edu (S.E.C.); cbecker@trinity.edu (C.B.B.)

**Keywords:** binge eating, food insecurity, psychological comorbidity, older adults

## Abstract

**Background/Objectives**: Evidence suggests that food insecurity (FI) is a risk factor for eating disorder (ED) symptoms, especially binge eating (BE), yet research focusing on the psychosocial effects among midlife/older women is lacking. Midlife/older women living with FI experience intersectional disadvantage, thus highlighting the need for an independent investigation of the cultural and contextual factors of this population. The current study examined the difference in psychological health and quality of life (QOL) among women living with BE and FI (BE + FI) versus FI without BE. **Method**: Female clients of a food bank, aged 50+ (*N* = 295; *M* age = 62.1 years, SD = 8.2) living with FI completed measures of BE and psychosocial comorbidities. The measures were provided in English and Spanish. **Results**: A multivariate analysis of covariance compared women living with BE and FI (BE + FI) versus FI without BE on outcomes related to mental health and wellbeing. Covarying for age, FI severity, and ethnicity, the results indicated that women living with BE + FI reported worsened anxiety, depression, ED-related psychosocial impairment, internalized weight stigma, and QOL versus women living with FI without BE (all *p*s < 0.001). Effect sizes ranged from small to medium to large. **Conclusions**: Midlife/older women living with BE + FI report poorer psychological health and QOL than those living with FI without BE, demonstrating a critical need for mental healthcare in this population. Innovative solutions—and likely a portfolio of interventional approaches with various entry points and delivery modalities—are warranted, if we are to make significant strides in addressing ED symptoms in this population.

## 1. Introduction

Food insecurity (FI) is defined as a household-level condition marked by an inability to consistently access nutritional and safe foods in socially acceptable ways [1,2]. The severity of FI is considered to be a spectrum in which households may range from high food security (i.e., regular access to sufficient food) to very low food security (i.e., disrupted eating patterns and extremely reduced food intake) [2]. FI is a worldwide health issue that impacts low-, middle-, and high-income nations like the United States [3]. In 2020, at least 60 million Americans (one in every five Americans) were recorded visiting food pantries, food banks, and other community food initiatives [4].

FI represents a significant social determinant of health (SDoH) barrier that negatively impacts physical health, mental health, wellbeing, and school/work [5,6,7]. Additionally, bourgeoning research has identified FI as a potential risk factor for eating disorder (ED) symptoms [8]. EDs are serious psychiatric illnesses that carry risks for significant health commodities such as osteoporosis, cardiac arrhythmia, and electrolyte abnormalities [9,10]. Claiming the lives of over 10,000 Americans every year, EDs have the second highest mortality rate of all mental health disorders [11,12]. Importantly, findings indicate that FI may comprise a risk factor for EDs, which challenges previously held stereotypes about who is at risk for EDs (i.e., thin, young, white, affluent, women/girls) [13]. The limited research conducted to date indicates that FI may be a risk for ED pathology via multiple etiological pathways.

For instance, individuals living with FI experience dietary restraint secondary to limited food availability in the home [14]. Notably, dietary restraint as a construct has been widely recognized to increase the risk for EDs among food-secure populations [15,16]. In FI populations, research indicates that ED risk from dietary restraint may be particularly elevated in the case of parents restricting their own food intake even when food is available so as to reserve food for children in the home [14].

Cyclical periods of more and less severe FI also can result in discrete and alternating periods of heightened food scarcity and availability. This, in turn, may result in periods of elevated consumption—even resembling or representing binge eating—when more food is available in the home, especially when subsequent meals are not guaranteed [17]. Indeed, research supports the association between FI and binge-eating (BE) severity and frequency, greater loss of control, and elevated rates of binge-eating disorder (BED) among adolescents and adults living with FI [18,19,20,21,22,23,24]. Thus, mounting data indicate that living with FI, including the cyclical patterns of scarcity/availability, likely increases the risk for ED behaviors.

FI and BE are also linked by a number of systemic issues that contribute to worsened health outcomes, including geographical barriers to healthy eating, and decreased healthcare access [25,26]. For instance, many FI households have limited access to high-quality grocery stores that sell affordable nutritious food (i.e., conditions found in food deserts [27,28,29]). FI households also often are located in “food swamps”, which are associated with easy access to food retailers (e.g., fast food restaurants, convenience stores) selling low-cost and highly palatable food (HPF; i.e., processed food high in salt, sugar, and fat [29]. Research investigating BE with animal models suggests that consumption of HPF can contribute to the development of BE. For instance, Boggiano and colleagues [30] found that some rats begin to BE when HPF is introduced into their diet even though they consumed normal amounts of food when their diet consisted of rat chow. HPF also has been linked to behaviors associated with BE, such as intense cravings and loss of control over consumption [31]. Thus, research supports that those who frequently consume these foods are at a higher risk of ED pathology.

Additionally, low-income individuals living with FI often lack regular access to healthcare. The luxury of preventive treatment, dietary education, and mental healthcare is not available to those who cannot afford to visit the doctor. Without access to mental health resources, individuals may turn to engaging in unhealthy coping mechanisms, such as BE [32]. Although engaging in such behaviors can offer a temporary sense of relief from stress, it ultimately leads to worse health outcomes over time.

To date, the majority of FI/ED research has focused on adolescents and younger adults, thus neglecting the potential risks related to ED symptoms for individuals in mid- or later life living with FI. Importantly, midlife may be a window of vulnerability for developing EDs, as women face a series of physical, hormonal, and psychosocial transitions. Menopause-related hormonal changes can trigger fluctuations in body shape, weight, and metabolism, which contributes to body dissatisfaction and unhealthy eating patterns. Furthermore, women in midlife often experience psychosocial transitions, such as becoming part of the “sandwich generation”, where they simultaneously care for aging parents and children in the home. These transitions can compound added stress and anxiety, leading to unhealthy coping mechanisms (e.g., emotional eating). Data show that 12–26% of midlife/older women engage in at least weekly BE (consuming an abnormally large amount of food in one sitting while simultaneously feeling out of control [33,34,35,36,37,38], meeting the frequency criterion for (BED). In the limited data examining ED pathology among midlife and older adults living with FI, BED was the most common ED behavior endorsed, and a more severe FI was correlated with a greater risk for BE [38]. Among these prior studies examining the frequency of ED behaviors among midlife and older women living with FI, 17–20% of women reported BE on at least a weekly basis [37,38]. Hooper and colleagues reported that 44.5% of midlife and older adults (68.5% female) reported BE in the past month, with no gender differences in the rates of reporting BE during this time frame [38].

As data are starting to emerge regarding the prevalence and frequencies of ED behaviors among midlife and older women living with FI and other significant SDoH barriers, gaps remain regarding the full clinical picture of individuals living with both BE and FI. In particular, gaps remain regarding the potential differential health burden of living with BE and FI, versus FI alone, in older populations living with intersectional disadvantage (older age, female gender, limited financial resources, minority ethnoracial identity [39]. Among younger, majority, and non-food insecure populations, BED is associated with myriad health consequences, including worse mental health status and quality of life (QOL). Limited data exist, however, on the psychosocial health correlates of BED when living with significant SDoH, such as FI. Thus, the purpose of the current study is to examine the differences in psychosocial health indices and quality of life (QOL) among midlife/older women living with BE and FI (BE + FI) versus FI without BE while controlling for specific established ED risk factors (age, racioethnic identity, and severity of FI). This comparison allows us to evaluate the potential additive effect of BE in the context of living with FI and other significant SDoH barriers (e.g., limited financial resources, lower levels of education), which comprise risk factors for health disparities broadly. We hypothesized that women living with BE + FI would report worse psychosocial health compared to those living with FI without BE.

## 2. Materials and Methods

### 2.1. Participants

The participants included women (*N* = 313) aged 50+ years (*M* = 62.1 years, *SD* = 8.2; range 50–94 years) who sought assistance at the San Antonio Food Bank (SAFB). Of note, the SAFB’s language regarding the people it serves has changed in recent years from “clients” to “neighbors” (i.e., the SAFB serves neighbors). As such, we use this terminology to reflect the preferred language of the SAFB. All women in this study identified as living with FI. The majority of participants (80%) self-identified their ethnicity as Hispanic/Latina. Most neighbors (66%) reported an annual household income of ≤USD 12,000; 31.2% reported graduating from high school. Additionally, over a third of individuals reported living with a disability (31%). See Table 1 for a breakdown of the sociodemographics by group.

### 2.2. Procedure

This study was conducted in collaboration with the SAFB and approved by the Trinity University Institutional Review Board (IRB). The SAFB provides service to 29 counties in Texas and serves over 100,000 neighbors per week. Locally, the child FI rate at the SAFB is 22.2%; data from the U.S. Department of Agriculture show that 15.5% of households in Texas are food insecure. Approximately 20% of people residing in San Antonio live below the official poverty line (U.S. Census Bureau, 2023) [40].

While food distribution is the main duty of the SAFB, the SAFB is also partnered with Workforce Solutions and Alamo Neighbor Services to assist with employment opportunities and federal benefits. Research assistants (RAs) approached individuals at the SAFB who were waiting for these services; wait times for services average around 30–50 min, which meant that interested neighbors could participate in the study without any disruption to their access to services at the SAFB. We note that we intentionally recruited individuals who were not waiting to receive food. RAs used standardized recruitment scripts (provided in both English and Spanish to accommodate neighbors’ language preferences) to inquire about a neighbor’s interest in study participation. Interested neighbors were given more detailed information. RAs followed the informed consent process and provided interested neighbors with a consent form; they had the choice to read this form independently or have a summary of each section read aloud by RAs.

After providing informed consent, the participants completed a self-report survey. Since literacy rates are lower among individuals with FI, all materials were altered to match a sixth-grade reading level. Given the high frequency of Spanish speakers in San Antonio, all scripts and measures were provided in English and Spanish. Any measures that were not already available in Spanish were translated into Spanish by two bilingual members of the research team. To verify that the meaning of each question remained the same in both English and Spanish, two other bilingual members of the research team back-translated all materials. A bilingual individual who grew up in San Antonio then reviewed the Spanish version and made small changes to reflect the local Spanish dialect spoken in San Antonio (see Becker et al. 2017, 2023 [18,41], for full details regarding measures modifications for reading level and Spanish language). Spanish-speaking RAs were also on-site throughout the entire data collection period.

Based on individual preference, participants were given the choice of either a paper or tablet version of the survey. For any participants who experienced difficulty reading the questionnaire, RAs read aloud survey items and response options. The survey took an average of 25 min to complete. In order to protect neighbor anonymity, all questionnaires were numerically coded in advance to organize the data without collecting identifying information. To further maintain confidentiality, the participants were asked not to include their names or any identifying information on the survey. Upon completion of the self-report survey, participants signed for a USD 8 gift card to the largest grocery store in the area. Afterward, RAs had the opportunity to debrief each participant and give them a copy of the consent form along with a list of free or low-cost mental health services.

#### Demographics

Participants self-reported their age, race and ethnicity, primary language spoken, annual household income, highest level of education, marital status, employment status, children in the household, disability status, and medical history. While waiting for services on-site, the participants also completed measures of FI, ED pathology, anxiety, depression, QOL, ED-related psychosocial impairment, internalized weight stigma, and body shame (see Table 2 for descriptives). We have conducted 3 previous studies in collaboration with the SAFB (*N* > 2000 participants since 2016) and found that this population has very low access to physicians and/or scales (which are a luxury item). As such, many neighbors have no way of knowing their weight. Thus, we opted to remove questions about height and weight out of data validity concerns.

### 2.3. Food Insecurity (FI)

The Radimer/Cornell Food Insecurity Measure (RCFIM [42,43]) was used. To assess FI, we used the 13-item RCFIM. Items in this measure are scored on a 3-point Likert scale (0 = not true; 1 = sometimes true; 2 = always true). A sample item includes “I worry about whether my food will run out before I get money to buy more”. Using responses from the RCFIM, the participants are classified into one of the following groups: (1) household FI, which is linked to food scarcity but not hunger; (2) individual FI, which involves an inability to obtain enough food for every member of the household because of insufficient resources; and (3) child-hunger household FI, where adults report having hungry children at home [43]. Child hunger in the home is considered the most severe level of FI because adults will generally try to protect children from hunger. Thus, the adults in the household are likely hungrier than the children. We reclassified the least severe group as “not food insecure” (NFI) rather than “food secure”. Despite some participants not meeting the full criteria for FI, all participants in this study are utilizing resources at the SAFB, indicating that they are living with some degree of FI. Kendall and colleagues [42] reported that the RCFIM has strong internal consistency. The internal consistency in our current sample was high (Cronbach’s α = 0.92).

### 2.4. Eating Disorder Pathology

The Eating Disorder-15 (ED-15 [44]) was used. We assessed ED pathology using the ED-15. This 15-item questionnaire measures the frequency of ED symptoms during the last week. Items are scored on a 7-point Likert scale (0 = not at all to 6 = all of the time). This measure includes two subscales, ‘Weight & Shape Concerns’ and ‘Eating Concerns’. The first ten items of the ED-15 measure eating attitudes; a sample item is ‘Over the past week, how often have I been preoccupied with thoughts of food and eating’. The last five items require open-ended responses and are only answered if participants have partaken in dysregulated behaviors, such as binge eating. We used self-reported BE episodes on the ED-15 for group categorization (i.e., BE + FI versus FI without FI). The ED-15 has been shown to have good clinical validity and reliability (current sample α = 0.95; [44]).

### 2.5. Anxiety

The Generalized Anxiety Disorder questionnaire (GAD-7 [45]) was used. We used the seven-item GAD-7 to assess the frequency of anxiety symptoms. This questionnaire asked participants to reflect on the preceding two weeks and rate their anxiety on a 4-point Likert scale (0 = not at all to 3 = nearly every day); higher scores indicate more significant anxiety symptoms. The GAD-7 is a well-validated and reliable measure of anxiety (α = 0.92). Internal consistency within our sample was excellent (α = 0.94).

### 2.6. Depression

The Personal Health Questionnaire Depression Scale (PHQ-8 [46]) was used. We employed the 8-item version of the PHQ to screen for depressive symptoms over the past 2 weeks. Participants were asked to rate how often they were bothered by problems such as feeling hopeless or depressed. Responses were rated on a 4-point Likert scale (0 = not at all to 4 = nearly every day), and higher scores indicate greater depressive symptoms. Research suggests that the PHQ-8 is a valid and reliable measure of depression (Cronbach’s α = 0.88). We used the PHQ-8 instead of the 9-item version (PHQ-9) because the PHQ-8 excludes the item inquiring about suicidal ideation (“Thoughts that you would be better off dead, or of hurting yourself”). The PHQ-8 and the PHQ-9 are highly correlated (r = 0.997) and have similar sensitivity [47]. Within our sample, internal consistency for the PHQ-8 was excellent (α = 0.92).

### 2.7. Quality of Life (QOL)

The EUROHIS-QOL-8 was used [48]. QOL was measured using the 8-item EUROHIS-QOL-8, which is adapted from the World Health Organization Quality of Life Scale (WHOQOL-BREF). We selected this measure to capture QOL, as it is significantly briefer than many QOL measures and, therefore, minimizes participant burden. Items on this scale measure the overall quality of life, energy, general health, self-esteem, daily living activity, finances, living conditions, and social relationships over the past two weeks. Items are scored on a 5-point Likert scale, with higher scores indicating better QOL. Internal consistency in the current sample was good (α = 0.85).

### 2.8. Eating-Related Psychosocial Impairment

The Clinical Impairment Assessment Questionnaire (CIAQ [49]) was used. We administered the CIAQ to measure ED-related psychosocial impairment over the past month. This 16-item questionnaire asks about the impact of exercising, eating habits, and feelings about weight/shape on various aspects of life (e.g., cognitive functioning, social engagement, and work performance) using a 4-point Likert scale (0 = not at all to 3 = a lot). The CIAQ score is summed; higher scores indicate greater impairment. The CIAQ has very good internal consistency and construct validity [49]; internal consistency in the current sample was excellent (current α = 0.98).

### 2.9. Weight Stigma

The Weight Self-Stigma Questionnaire (WSSQ [50]) was used. We used the WSSQ to measure internalized weight stigma. The WSSQ consists of 10 items on a 5-point Likert scale (1 = completely disagree to 5 = completely agree). For this study, we used the total score; higher scores indicate greater weight self-stigma. Similar to our previous studies with populations living with FI [18,19], we modified questions to improve comprehension for individuals with lower reading levels. Two items from the original measure (twelve items) were removed due to the complexity of the questions, and we changed the wording of “weight problems” and “overweight” to “fat” (e.g., “People discriminate against me because I’m fat”). This modified measure has had excellent internal validity in prior work (α = 0.94 [41]); internal consistency in our sample was high (α = 0.94).

### 2.10. Body Shame

The Body Image Shame Scale (BISS [51]) was used. We used the BISS global score to assess the overall body shame experienced by participants. The BISS is comprised of 14 items, which can be divided into 2 subscales (internalized and externalized body shame). Factor analyses have demonstrated that the BISS can be scored as a global factor or as separate subscales representing distinct factors that underlie this higher-order global factor on body image shame. Psychometric properties for both the global score and the subscales are strong [51]. Thus, to minimize the number of tests, we used the global score to capture body shame in this sample. Global internal consistency was high in the current sample (α = 0.98).

#### Analytic Strategy

Descriptive statistics captured sociodemographic variability, including established ED-risk variables of FI severity, age, and ethnicity. Groups were split based on the frequency of BE episodes over the past week; those who reported a ≥1 BE over the past week on the ED-15 (*n* = 57; 20.1%) were categorized in the BED + FI group, while those who denied any BE in the past week on the ED-15 (*n* = 227; 79.9%) were categorized as the FI group. To examine potential between-group differences in all of the health indices while accounting for multiple dependent variables, we conducted a multivariate analysis of covariance (MANCOVA), covarying for participant age, race/ethnicity, and FI severity. Levene’s test of equality of error variances examined the error variance of the dependent variable across groups. Box’s M Test and Pillai’s Trace statistics were used to assess the variance–covariance matrices and account for unequal matrices when evaluating assumptions.

## 3. Results

Levene’s test of equality of error variances indicated that all dependent variables were of equal variances across groups, except the CIAQ, which measures ED-related impairment. Box’s M Test of equality of covariance matrices (including intercept + age + ethnoracial group + FI severity + BE group) was significant (*p* = 0.001), suggesting that there may be unequal covariance matrices. Because Box’s M is oversensitive in large samples, such as ours, we examined Pillai’s Trace as a more robust test of true between-group differences. Pillai’s Trace was significant for our independent variable (*p* < 0.001), suggesting a true difference between the BE + FI and FI groups.

Overall, women living with BE + FI reported higher anxiety (*p* < 0.001) and depressive symptoms (*p* < 0.001), greater ED-related psychosocial impairment (*p* < 0.001), poorer QOL (*p* < 0.001), higher internalized weight stigma (*p* = 0.001), and greater body image shame (*p* < 0.001) than women living with FI without BE when covarying for age, ethnicity, and FI severity. Between-group effect sizes ranged from small to medium to large. Regarding covariates, older age was significantly related to lower anxiety (*p* < 0.001) and depression symptoms (*p* = 0.023), less ED-related impairment (*p* < 0.001), and lower body image shame (*p* = 0.006). Greater severity of FI was related to higher anxiety (*p* < 0.001) and depressive symptoms (*p* < 0.001), poorer QOL (*p* < 0.001), and more ED-related impairment (*p* = 0.028) and body shame (*p* = 0.031). Ethnicity was not significantly related to any of the outcome variables. See Table 3 for results.

## 4. Discussion

The purpose of this study was to investigate the potential additive psychosocial comorbidity of living with BE in the context of FI (BE + FI) versus FI without BE among older women. Importantly, *all* women in the study reported significant SDoH barriers in addition to living with FI (e.g., low annual household income, lower levels of education) that did not differ between groups. As such, the primary aim of this study was to begin identifying the potential additive psychosocial burden that living with BE contributes by comparing women with similar SDoH barriers and sociodemographic characteristics.

The results indicated that approximately 20% of older adult women (aged 50+) living with FI reported BE on at least a weekly basis. Consistent with our hypotheses, older women living with BE + FI reported greater anxiety and depressive symptoms, ED-related impairment, internalized weight stigma, and body shame, as well as poorer QOL when covarying for age, racioethnic identity, and FI severity. Thus, living with BE in the context of FI is associated with elevated psychosocial comorbidity, above and beyond that experienced by older women who experience similar and significant SDoH barriers. These results shed light on factors relevant to BE and SDoH in two primary ways: (1) the additive psychosocial burden of BE and FI, compared to that of living with FI in the absence of BE, and (2) the cultural and contextual considerations needed for developing and expanding services for older women living with this complex clinical picture in an environment without easy access to care.

First, the results highlight the psychosocial burden faced by women living with FI and BE, demonstrating a multifaceted need for mental healthcare in this population. Notably, given the intersectional disadvantage of women in our sample (i.e., female, older age, minoritized ethnicity, living with FI, living with lower financial resources, and education), it is critical that we first consider the important cultural and social factors potentially amplifying these additive psychosocial burdens of living with BE in the context of FI, including but not limited to, systematic forms of oppression. For example, due to new Texas laws targeting those who are undocumented, individuals may experience heightened levels of emotional distress and may be more hesitant to go to places offering services, further exacerbating their emotional distress. This exacerbated emotional distress may then interact with financially based dietary restraint to make undocumented individuals and their loved ones even more susceptible to engaging in BE behaviors [52] and the additive psychosocial burden of BE in the context of FI, as seen in this sample. Building on this example of cultural and contextual relevance in identifying risk factors for diverse populations, future research is needed to better disentangle the nuances of living with FI and ED pathology so as to advance the field in ways that promote inclusivity and expand our conceptualizations of those who are at risk for EDs and how and why. This is especially important when considering the context of systemic oppression and historical discrimination in the medical system, which has resulted in earned medical mistrust by many minoritized and marginalized populations.

Second, we need a deepened understanding of the potential harms of living with ED pathology (e.g., BE) in the context of facing significant SDoH barriers to recovery in order to formulate innovative approaches for improving health equity. In recognizing that many of these factors are maintained on the institutional level, it is imperative that we conceptualize, develop, build, and advocate for multi-level interventions, which will very likely involve advocacy work in health and resource policy. Innovative solutions—and likely a portfolio of multi-level interventional approaches with various entry points and delivery modalities—are warranted if we are to make significant strides in improving mental health equity. Specifically, target system-level interventions to not isolate or target the individual living with significant SDoH barriers. In other words, it is important that we conceptualize these psychosocial burdens differently than we do for individuals living with sufficient resources. It is also critical to approach our conceptualization of psychopathology through a lens of understanding the lives of individuals facing SDoH barriers to health equity, as some behaviors historically seen as disordered in the context of privilege may serve as survival behaviors in the context of poverty (e.g., food restriction). Ultimately, this mindset is needed to ensure that we do not pathologize living with SDoH barriers by putting the onus on the individual rather than the systems of oppression in which they are living. This new conceptualization will, in turn, pave the way for better and more inclusive interventions.

Longitudinal research that evaluates the prospective impacts of living with BE and FI using a multi-method assessment is also urgently needed if we are to identify and understand causal pathways. Further, the use of mixed-methods research would assist in examining and better understanding systematic, cultural, and individual barriers and facilitators to diagnosis and treatment, as well as how these various factors interact. Longitudinal mix-methods research is critical if we want to understand the bidirectional relationships that undoubtedly need to be investigated. For instance, BE may trigger depression, particularly if out-of-control eating worsens FI. Yet, depression may lead to a search for emotion regulation strategies, which may include BE. These two relationships might exist in the same person, again creating a cascade of psychosocial distress.

Future research will additionally need to be focused on understanding stigma and how stigma impacts the chronicity of BE and other psychosocial comorbidities among older women living with FI. We will likely need to develop system-level interventions, with consideration for cultural and contextual factors, such as environmental circumstances that impede or promote remission of BE in the context of living with FI. Future research also will need to investigate preventive programming at the system level (i.e., not exclusively targeting the individual living with FI for treatment without considering the lived environment, which reflects a longstanding history of systems of oppression).

Not everyone who lives with FI goes on to develop BE. Recent research suggests that temperamental factors may influence the emergence of various mental health concerns, including EDs [53]. Longitudinal research will also play a much-needed role in determining how temperament interacts with SDoH so that targeted interventions for individuals can also be developed, given that systemic-level interventions can take time to implement, particularly when government is resistant to this level of intervention.

Although further research is critically needed to inform both policies and clinical practice, a few common-sense suggestions are in order. Those working with older women living with FI should screen for BE, as older women with BE have reported feeling their distress about their eating is both invisible and unheard [34]. De-stigmatizing pathological eating behaviors and offering validation and empathy are critical first steps in providing support. For those who treat EDs, all patients should be screened for FI, since patients can experience shame around FI and fail to voluntarily disclose their difficulty procuring adequate food. Asking about FI normalizes it and makes it easier for patients to discuss their challenges. Given the critical role of dietary restraint in the onset and maintenance of EDs, all providers also should have plans in place (e.g., supplements; food; gift cards to buy food; and resources for facilitating access to food banks) to help patients living with FI become more food secure.

### Limitations

The limitations of the current study include its cross-sectional design, which uses self-report data. Ideally, future research should capture BE symptoms and psychosocial comorbidities using multi-faceted assessments and longitudinal designs. Given the cross-sectional nature of this study, we cannot infer the chronology of psychosocial symptom onset or maintenance. It could be that living with BE and FI increases the risk for other mental health symptoms. Alternatively, facing psychological symptoms, such as depression, increases the likelihood of developing BE symptoms. Future research is needed to disentangle the risk profile of living with FI for older populations to identify potential avenues for preventive, multi-level interventions. The ED-15, while brief and minimizing participant burden, only captures BE in the past week. Given the cyclical nature of food insecurity in some populations, this could correspond to cyclical patterns of ED symptom fluctuation. Participants in this study, however, were not presenting to the food pantries (i.e., for food distribution) but to Neighbor Services (e.g., applying for or renewing federal benefits). Thus, they were not presenting for food distribution at the time of study participation. The prevalence of BE over the past week in this study (20%) matches past studies that capture BE frequency over longer periods of time (e.g., [37], suggesting that this may reflect a more longstanding frequency of BE behaviors. Additionally, because we included all older women presenting for services at the SAFB, this resulted in unequal sample sizes between the BE + FI and FI groups. The use of multivariate analyses with covariate adjustments reduces the potential influence of unequal sample sizes; Pillai’s Trace further supported the interpretation that differences between groups existed. Future research using selective sampling strategies could remedy sample size limitations.

## 5. Conclusions

Living with FI is associated with an increased risk for myriad mental health symptoms, including ED pathology [18,19,24]. This study demonstrates that living with BE in the presence of FI worsens the psychosocial burden experienced by older women above and beyond that associated with FI and related SDoH. Mixed-methods longitudinal research is critically needed to understand causal pathways and to begin to create the foundational knowledge that will be crucial to the development of both system-level and individual-level preventative and treatment interventions. Given the role of SDoH in mental health disparities, innovative approaches with multi-level interventions will likely be needed in order to improve health equity for populations living with FI and ED pathology, including BE. Ultimately, it is time to overcome the notion that EDs are disorders of wealth. Instead, we need to recognize the intersecting nature of EDs and SDoH and strive for more inclusivity and health equity considerations in our research and intervention development (e.g., multi-level interventions).

## Figures and Tables

**Table 1:** Participant sociodemographic data.

Demographics	FI Only *n* = 250	FI + BE *n* = 63
	*M* (SD) or *N* (%)	*M* (SD) or *N* (%)
Age	62.2 (8.1)	61.5 (9.1)
Ethnicity		
Latino/Hispanic	203 (81.2%)	46 (73%)
Black/African American	8 (3.2%)	3 (4.8%)
White/Caucasian	28 (11.2%)	11 (17.5%)
Other/Multiple *	11 (4.4%)	3 (4.8%)
Primary language spoken		
English	139 (55.8%)	38 (60.3%)
Spanish	73 (29.3%)	17 (27%)
English and Spanish	37 (14.9%)	8 (12.7%)
Food Insecurity		
Not food insecure	17 (6.8%)	---
Household FI	26 (10.4%)	1 (1.6%)
Individual FI	145 (58%)	41 (65.1%)
Child hunger	62 (24.8%)	21 (33.3%)
Education		
No or some grade school	30 (12%)	9 (14.2%)
Finished grade school	16 (6.4%)	5 (7.9%)
Some high school	52 (20.8%)	11 (17.5%)
High school/GED	80 (32%)	18 (28.6%)
Some college or technical	54 (21.6%)	16 (25.4%)
Bachelor+	18 (7.2%)	4 (6.4%)
Annual household income		
<USD 10,000	108 (45.8%)	30 (49.2%)
USD 10,000–USD 35,000	112 (47.6%)	30 (49.2%)
USD 35,000–USD 90,000	15 (6.2%)	---
USD 90,000+	1 (0.4%)	1 (1.6%)
Marital status		
Single	66 (26.4%)	12 (19%)
Married/living with partner	66 (26.4%)	18 (28.6%)
Divorced or separated	68 (27.2%)	22 (34.9%)
Widowed	50 (20%)	11 (17.5%)
Children under 18 in the household	70 (28%)	17 (27%)
Living with disability	78 (31.2%)	24 (38.1%)
Diabetes	131 (52.4%)	36 (57.1%)

Notes. * Some racioethnic groups were collapsed into ‘other’ due to small sample sizes, including Native American (*n* = 1) and multiple races.

**Table 2:** Descriptive statistics for dependent variables.

Measures	FI Only *M* (SD)	FI + BED *M* (SD)
Depression (PHQ-9)	7.6 (6.4)	11.7 (5.8)
Quality of Life	0.34 (0.58)	0.79 (0.78)
Internalized Weight Stigma (WSSQ)	25.7 (5.8)	21.7 [5]
Clinical Impairment (CIAQ)	21.6 (10.2)	27.2 (11.5)
Body Image (BISS)	6.9 (10.8)	19.4 (14.5)
Anxiety (GAD-7)	12 (15.9)	30.4 (16.8)

**Table 3:** MANCOVA Results.

		Measures	F	Sig.	Partial Eta Squared
Covariates	Age	Anxiety	15.860	<0.001	0.053
		Quality of Life	1.027	0.312	0.004
		Internalized Weight Stigma	2.232	0.136	0.008
		Depression	5.218	0.023	0.018
		Clinical Impairment	12.527	<0.001	0.042
		Body Image	7.564	0.006	0.026
	Ethnicity	Anxiety	0.204	0.652	0.001
		Quality of Life	0.072	0.788	0.000
		Internalized Weight Stigma	0.018	0.893	0.000
		Depression	0.157	0.692	0.001
		Clinical Impairment	0.011	0.917	0.000
		Body Image	0.456	0.500	0.002
	FI Severity	Anxiety	15.769	<0.001	0.053
		Quality of Life	38.883	<0.001	0.120
		Internalized Weight Stigma	0.091	0.763	0.000
		Depression	19.158	<0.001	0.063
		Clinical Impairment	4.900	0.028	0.017
		Body Image	4.693	0.031	0.016
Fixed Factor	BED + FI vs. FI	Anxiety	14.060	<0.001	0.047
		Quality of Life	15.260	<0.001	0.051
		Internalized Weight Stigma	10.834	0.001	0.037
		Depression	24.141	<0.001	0.078
		Clinical Impairment	48.859	<0.001	0.147
		Body Image	53.932	<0.001	0.160

## Data Availability

The dataset generated and/or analyzed during the current study is not publicly available but is available from the corresponding author upon reasonable request.

## Abbreviations

BE	Binge eating
BED	Binge-eating Disorder
ED	Eating Disorder
ED	15-Item Eating Disorder Pathology Questionnaire
FI	Food Insecurity
NFI	Not Food insecure
IRB	Institutional Review Board
QOL	Quality of Life
RCFIM	Radimer/Cornell Food Insecurity Measure
GAD-7	7-Item Generalized Anxiety Disorder Questionnaire
PHQ-8	8-Item Personal Health Questionnaire
EUROHIS-QOL-8	8-Item European Health Interview Survey-Quality of Life
CIAQ	Clinical Impairment Assessment Questionnaire
WSSQ	Weight Self-Stigma Questionnaire
BISS	Body Image Shame Scale
MANCOVA	Multivariate Analysis of Covariance
SDoH	Social Determinants of Health
